# Systematic review of the oral manifestations produced by the SARS-CoV-2 vaccine

**DOI:** 10.4317/jced.60688

**Published:** 2023-07-01

**Authors:** Carmen López-Carriches, Tomás Santana-Torres, Ricardo Bahram-Taheri, Mª Isabel Leco-Berrocal

**Affiliations:** 1 Associate Professor. Department of Dental Clinic Specialties. School of Dentistry. Universidad Complutense de Madrid. Spain; 2 Doctor of Dental Surgery. DDS. Collaborator. School of Dentistry. Universidad Complutense de Madrid. Spain; 3 Assistant Professor. Department of Dental Clinical Specialties. School of Dentistry. Universidad Complutense de Madrid, Spain

## Abstract

**Background:**

To combat the coronavirus pandemic different vaccines have been developed. However, diverse adverse effects have been reported due to their use, including oral manifestations. Our objective is to review the existing bibliography to analyze what complications these vaccines have caused in the oral cavity.

**Material and Methods:**

A bibliographic search was conducted by two independent reviewers (TS and CL), in parallel in 6 electronic databases (PubMed, Scopus, Cochrane, Google Scholar, LILACS, BioMed Central). A total of 22 articles were analyzed.

**Results:**

The most frequent adverse effect was oral lichen planus, with a higher prevalence in women and after the Pfizer mRNA BNT162b2 vaccine.

**Conclusions:**

These complications are minor and resolve with treatment, so the benefit of the use of vaccines outweigh the potential risks associated with these.

** Key words:**Covid-19 vaccine, oral lesions, oral manifestations, SARS-CoV-2 vaccine, oral symptoms.

## Introduction

The pandemic produced by SARS-Cov-2 has been a challenge for the world population, largely mitigated by the appearance of vaccines which have significantly reduced the incidence.

Anti-SARS-CoV-2 vaccines approved by the FDA (United States Food and Drug Administration) and EMA (European Medicines Agency) are messenger RNA (mRNA) vaccines. The most used are BNT162b2 Comirnaty (Pfizer/BioNTech, New York, NY, USA) and mRNA-1273 Spikevax (Moderna, Cambridge, MA, USA). The mRNA encoding the S protein is encapsulated in lipid nanoparticles. Nucleic acid vaccines introduce mRNA or DNA encoding the SARS-CoV-2 spike protein into cells, inducing them to produce antibodies (1).

In addition, viral vector vaccines are used. These vaccines use a chemically weakened virus (eg, adenovirus) to insert the code for SARS-CoV-2 antigens into cells. The two main viral vector vaccines are Jcovden Ad26.COV2.S (Janssen, Johnson&Johnson New Brunswick, NJ, USA) which is produced with human adenovirus carrying protein S. VaxzevriaChAdOx1-S, and AstraZeneca (Cambridge, UK) which includes non-replicating chimpanzee adenovirus carrying protein S. Another vaccine used is Covilo/BBIBP-Corv (Sinopharm Beijing, China) (2).

In this context, and taking into account that all pharmacological treatments have risks of producing adverse effects, our objective is to review the existing bibliography and determine the appearance of oral manifestations caused by vaccines against SARS-Cov-2, classifying these oral adverse effects according to epidemiological data and clinical manifestations.

## Material and Methods

A bibliographic search was conducted by two independent reviewers (TS and CL), in parallel in 6 electronic databases (PubMed, Scopus, Cochrane, Google Scholar, LILACS, BioMed Central). Publications in English and Spanish were reviewed using the following keywords: “Sars-cov-2 vaccine”, “Covid-19 vaccination”, “oral lesions” “oral complication”, “oral manifestation”. A total of 1236 articles were obtained from the different databases.

All identified articles were reviewed independently, including those that referred to the establishment of oral lesions after vaccination against SARS-CoV-2, including a total of 52 articles. For the management of bibliographic citations, ZOTERO program was used. A total of 42 articles were analyzed after deleting duplicates. After a qualitative review, 9 of the articles were not accessible in full format, 5 made exclusive reference to the oral manifestations produced by Covid-19 and not by the vaccine, 4 made no reference to oral manifestations and 5 were reviews of the literature, leaving us with a total of 22 articles.

As inclusion criteria, all patients were over 18 years of age, had received the COVID-19 vaccine and presented adverse oral manifestations after the administration of the vaccine. Literature reviews and clinical cases of oral manifestations caused by the disease and not by the vaccine were excluded.

## Results

The 22 articles analyzed in this review (Table 1- Table 1 cont.-1), include a total of 39 cases in which the oral lesions manifested by the patients could be directly related to the SARS-Cov-2 vaccine, since they did not present any other etiological agent.

**Table 1:**
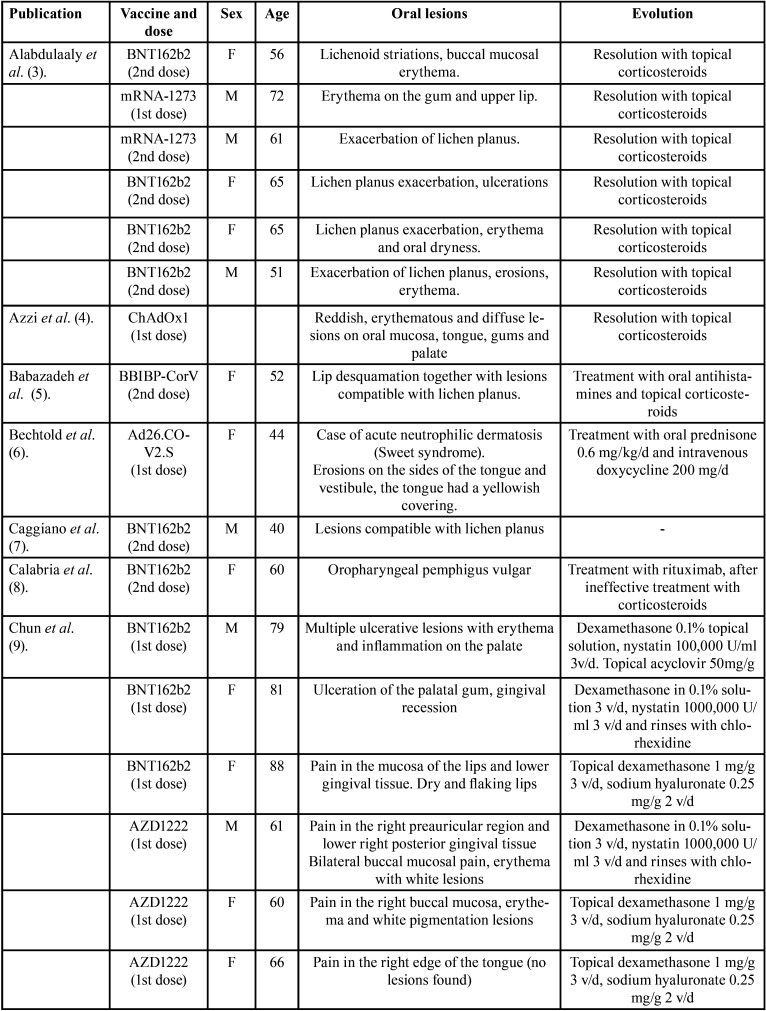
Articles included.

**Table 1 cont.**
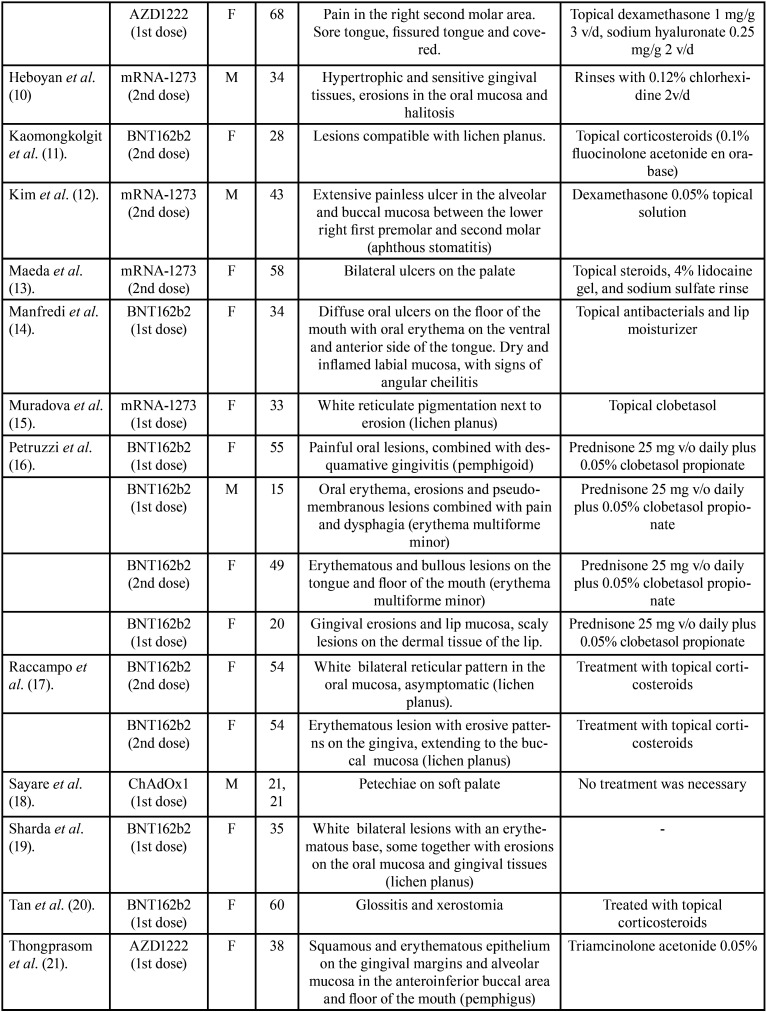
Articles included.

**Table 1 cont.-1:**
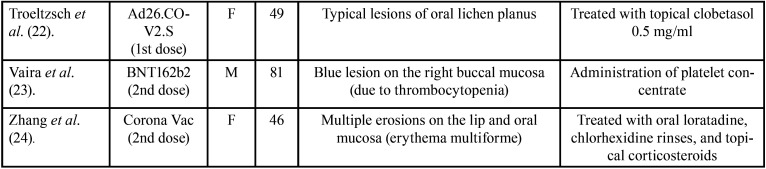
Articles included.

Only one of the articles (4) reported neither age nor sex. We found that 71.05% are women (n:27) and the remaining 28.94% men (n:11). In this line, the age range in the male sex is between 15 and 81 years old, with an average of 48.25 years. The average age is slightly higher in the female sex, reaching 56.7 years, with a minimum age of 20 and a maximum of 88. Therefore, the mean age of all cases is 52.66.

In relation to the type of vaccine, the one that produces more cases of oral manifestations is Pfizer’s BNT162b2 with 20 (55.55%) followed by mRNA-1273 (Moderna) with 6 cases (16.66%), AZD1222 (Astrazeneca) with 5 cases (13.88%), ChAdOx1 (Jenner) with 3 cases (8.33%), Ad26.COV2.S (Janssen) with 2 cases (5.55%) and finally Corona-Vac (Sinovac) and BBIBP-CorV (Sinopharm) with 1 case each (2 .77%). Regarding the dose, in 21 patients the adverse effects occurred after the first dose of the vaccine (55.26%); distributed as follows: BNT162b2 (PFIZER): 9, mRNA-1273 (MODERNA): 2, AZD1222 (Astrazeneca): 5, Ad26.COV2.S (JANSSEN): 2, ChAdOx1 (JENNER): 3, and in 17 patients occurred after receiving the second dose (44.73%); BNT162b2 (PFIZER): 11, mRNA-1273 (MODERNA): 4, BBIBP-CorV (sinopharm): 1, Corona-Vac (Sinovac): 1.

When classifying the lesions described, it is worth noting the appearance of lesions compatible with oral lichen planus; typical reticulated white plaques (Wickham’s striae) next to areas of erosion in some presentations. These lesions were present in 16 cases, 8 of them confirmed with histopathological diagnosis by biopsy (3,5,7,9,11,15,17,19,22). We also found nonspecific manifestations such as erythematous lesions, glossitis, halitosis, fissured tongue, ulcers, petechiae, sensitive gums, and xerostomia. Other lesions where found with less prevalence: 3 cases of erythema multiforme minor (16,24), 2 cases of pemphigus (8,21), 1 of pemphigoid (16), 1 aphthous stomatitis (12), 1 case of acute neutrophilic dermatosis (6) and 1 hemangioma due to thrombocytopenia (23).

## Discussion

The oral cavity is not exempt from complications after the COVID-19 vaccine. Riad *et al*. (25), studied the complications reported by drug agencies from different countries and found 128 oral adverse effects, 0.872% being oral paresthesia, followed by lip swelling (0.872%), ageusia (0.722%), hypesthesia (0.648%) and inflammation of the tongue (0.628%). These were more prevalent in older women. Also, it has been reported to be more prevalent after the first dose, especially vaccines based on messenger RNA. Adverse effects were more frequent than with the flu vaccine.

But the most frequently reported adverse effects are lichenoid lesions. Alharbi *et al*. (26), found that vaccinated patients had thousand times more odds of developing oral lichen planus compared to non-vaccinated patients.

On the other hand, Hertel *et al*. (27), carried out a retrospective analysis of patients vaccinated against Covid-19, to determine the risk of suffering from oral lichen planus as a consequence of the vaccine, with the percentage of patients who develop it of 0.027%. Demonstrating that the risk of vaccination producing these lesions is very low.

Joseph *et al*. (28), reviewed the treatments administered for oral manifestations after vaccination, evidencing that these cases were resolved by applying the conventional treatment with a good prognosis.

## Conclusions

Dentists must be aware of the adverse effects that the COVID-19 vaccine can have on the oral cavity in order to treat the possible complications the patient may manifest. However, these complications do not outweigh the benefit of COVID-19 vaccines in the prevention of the disease.
